# Patient Perceptions and Quality of Life in Pacemaker Recipients

**DOI:** 10.19102/icrm.2021.121103

**Published:** 2021-11-15

**Authors:** Maria Polikandrioti

**Affiliations:** ^1^Department of Nursing, University of West Attica, Athens, Greece

**Keywords:** Cardiac device, permanent pacemaker, quality of life, Short Form–36

## Abstract

Quality of life (QoL) reflects the multidimensional impact of a clinical condition and its treatment on patients’ daily lives. Although permanent cardiac pacemakers (PPMs) have made a significant contribution to the improvement of patients’ QoL, patients’ perceptions remain crucial after implantation. Hence, the present study was carried out to explore the QoL and the associated perceptions of PPM patients. A total of 150 PPM patients were enrolled. Data were collected using the Short Form–36 (SF-36) health survey, which also included patients’ characteristics. The statistical significance level was set at p < 0.05. The physical health score as measured by SF-36 was 42.9 ± 19.5 points, and the mental health score was 54.1 ± 26.6 points. Study participants had higher scores in emotional well-being (66.5 ± 18.8 points), and the lowest scores were in physical functioning (22.5 ± 10.7 points). The level of information about PPM was associated with physical role (p = 0.005), emotional role (p = 0.004), emotional well-being (p = 0.001), social functioning (p = 0.002), and general health (p = 0.001). Attendance at follow-up was associated with physical role (p = 0.015), emotional role (p = 0.014), social functioning (p = 0.003), and general health (p = 0.016). The belief that the device prevents disease deterioration was associated with physical role (p = 0.005), emotional role (p = 0.001), energy/fatigue (p = 0.010), emotional well-being (p = 0.004), social functioning (p = 0.001), pain (p = 0.005), and general health (p = 0.001). Dependency on the device was associated with energy/fatigue (p = 0.006), emotional well-being (p = 0.001), and social functioning (p = 0.002). Social difficulties due to the device were associated with emotional well-being (p = 0.001), social functioning (p = 0.001), pain (p = 0.001), and general health (p = 0.004). Family support was associated with emotional role (p = 0.023) and general health (p = 0.036), while pain was associated with information about the family (p = 0.001). In conclusion, the present findings regarding factors associated with QoL provide key opportunities for interventions aimed at facilitating positive adjustments after PPM implantation.

## Introduction

In the last several decades, the rapid growth of the medical device industry along with population aging has led to increased implantation rates of permanent cardiac pacemakers (PMs).^1^ This promising innovation in cardiac management has been widely used for arrhythmia therapy.^2,3^ According to global estimates, 1.25 million PPMs are implanted annually, which is anticipated to increase within the next few years.^4^ The upward trend in implantations is not a recent issue. In greater detail, the rate of PPM implantations in the United States increased by 45% over 16 years (1993–2008).^5,6^ In 2016, 500,000 PMs were implanted in Europe, with the rate of PM implantations being almost fourfold higher in the European Union member countries than in the non–European Union member countries (759% vs. 198%). Within Europe, the most active area has been Western Europe, with 1,174PM implantations per one million people.^7^

Given these global trends, a great deal of attention has been paid to the impact of pacing therapy on patients’ quality of life (QoL).^1^ Interestingly, device implantation has evolved from a life-saving therapy to one aimed at improving QoL.^8^ Shortly after device implantation, PPM recipients enjoy alleviation of symptoms such as chest pain, dyspnea, and fatigue.^2,3^ Even so, cardiac device implantation is not a single intervention but an ongoing treatment that requires follow-up visits for pacing settings.^9^ Therefore, evaluation of QoL has become a constant process.

From a philosophical perspective, QoL is considered as a person’s well-being, referring to the individual’s biographical and personal characteristics. From a societal perspective, maintaining QoL on the individual level creates stability and equality among the members of society as a whole. From the patient’s perspective, QoL reflects what actually happens in their daily lives and indicates the gap between their hopes and expectations. From a clinical perspective, QoL provides essential information to health professionals when planning patient-centered care practices.^10^

In recent times, patients are increasingly encouraged to take up an active role in managing their health by expressing their concerns and options and by participating in medical decisions. The implantation of a PM per se as a technological advance has an impact on patients’ perceptions related to their willingness to be involved in their care.^11^

Therefore, the notable aspect is the potential influence of patients’ perspectives on QoL. Importantly, this evaluation will guide clinicians when planning interventions that optimize patients’ ability to encounter implantation.

Hence, the objective of this study was to explore the QoL and the associated perceptions of PPM patients.

## Materials and methods

### Study population

In the present study, 150 outpatients with PPMs were enrolled. By means of convenience sampling, PPM patients who visited the cardiology outpatient department for periodic and scheduled follow-ups were invited to participate in the study. The criteria for inclusion in the study were: (1) PPM implantation; (2) ability to read and write the Greek language fluently; and (3) adequate follow-up. Meanwhile, patients (1) with a history of mental illness or other additive chronic organic diseases; (2) with implantable cardioverter-defibrillators; (3) lacking the cognitive ability to answer the questionnaires; or (4) presenting symptoms such as dyspnea, weakness, or fatigue at the time the instrument was applied were excluded.

### Procedure

PPM patients who agreed to participate in the study were invited to a private room to fill in the questionnaires, which ensured privacy and safety. The process of filling out the questionnaires lasted between 15 and 30 minutes and took place after patients had completed their follow-up in the outpatient clinic.

#### Ethical considerations.

The study was approved by the medical research ethics committee of the hospital. Written informed consent for inclusion was obtained from all patients after an explanation of the purpose and procedure of the study was given. Participation was on a voluntary basis and anonymity was preserved. Furthermore, all participants were informed of their rights to refuse or discontinue their participation. All procedures were performed in accordance with the ethical standards of the institutional and/or national research committee and with the 1964 Declaration of Helsinki and its later amendments or comparable ethical standards.

### Data collection

Data were collected using the Short Form–36 (SF-36) health survey scale. Data also included patients’ perceptions about their pacing therapy.

### Measurement of the quality of life

The SF-36 scale was used to assess patients’ QoL. The SF-36 assesses both physical and mental health. It consists of 36 questions covering eight dimensions: physical functioning, physical role, physical pain, general health, energy/fatigue, social functioning, emotional role, and emotional well-being. The respondents answered the questions on Likert-type scales. The scores assigned to the questions were summed up separately for the questions that evaluated the eight dimensions. Higher scores indicate a better QoL.^12^

### Statistical analysis

Categorical data are presented as absolute and relative frequencies (%), while continuous data are presented as median and interquartile range values as they did not follow the normal distribution (tested with the Kolmogorov–Smirnov criterion and graphically with Q–Q plots and histograms). Nonparametric Mann–Whitney U and Kruskal–Wallis tests as well as Spearman’s rho correlation coefficient were used to examine the association between patients’ QοL and characteristics.

In addition, multiple linear regression was performed to estimate the effect of patients’ characteristics on their QοL. Results are presented as β regression coefficients and 95% confidence intervals. The observed level of 5% was considered statistically significant. All statistical analyses were performed using the Statistical Package for the Social Sciences version 22 (IBM Corporation, Armonk, NY, USA).

## Results

### Sample description

Of the 150 patients with PPMs, men accounted for 55% of the study population, and the mean age of the sample studied was 62.8 ± 10.4 years. In terms of patients’ perceptions, 35.3% of the sample said they were well informed about their PPM therapy, while 40.7% stated that their family was well informed. Furthermore, 48% reported having a supportive family, while the majority of them reported having very good relationships with their nursing and medical staff (76% and 72.7%, respectively). Furthermore, 28.7% reported that they regularly attended the scheduled follow-up visits, 29.3% believed that they were highly dependent on the implanted cardiac device, 15.3% expressed the belief that the implanted cardiac device would prevent disease deterioration, and 60.7% reported that they did not experience any social difficulties due to the device **(Table 1).**

### Patient’s quality of life

**Table 2** presents results regarding patients’ QoL. Patients had higher scores in emotional well-being (66.5 ± 18.8 points) followed in descending order by by pain (63.8 ± 28.5 points), social functioning (61.7 ± 27.0 points), energy/fatigue (57.1 ± 20.1 points), emotional role (38.9 ± 45.2 points), physical role (35.4 ± 41.1 points), and physical functioning (22.5 ± 10.7 points). The general health SF-36 score was 48.0 ± 20.1 points. Also, the physical health score was 42.9 ± 19.5 points and the mental health score was 54.1 ± 26.6 points.

### Factors affecting patients’ quality of life

**Tables 3 to 5** present the association between patients’ characteristics and their QoL. Physical functioning **(Table 3)** was found to not be statistically significantly associated with any characteristic. Physical role **(Table 3)** was statistically significantly associated with the degree of information about PPM therapy (p = 0.005), whether the patient regularly attended the scheduled follow-up (p = 0.015), and whether the patient believed that the device prevents disease deterioration (p = 0.005). Additionally, older patients had a worse physical role (rho = −0.230). Patients who said they were well informed about PPM therapy (median: 50), those who regularly attended their follow-up (median: 50), and those who did not believe that their device prevents disease deterioration (median: 50) had a better physical role.

Regarding the emotional role **(Table 3)**, it was statistically significantly associated with the degree of information about PPM therapy (p = 0.004), family support (p = 0.023), whether the patient regularly attended the scheduled follow-up (p = 0.014), and whether the patient believed that the device prevents disease deterioration (p = 0.001). Additionally, older patients had a worse emotional role (rho = −0.220). Patients who were well informed about PPM therapy (median: 67), patients whose family was very supportive (median: 33), patients who regularly attended the scheduled follow-up (median: 67), and patients who did not believe that the device prevents disease deterioration (median: 33) had a better emotional role. Meanwhile, energy/fatigue **(Table 4)** was found to be statistically significantly associated with dependency on the device (p = 0.006) and whether participants believed that the device prevents disease deterioration (p = 0.010). More specifically, patients who did not feel dependent on the device (median: 60) and those who did not believe that the device prevents disease deterioration (median: 65) had a better QoL.

Emotional well-being **(Table 4)** was statistically significantly associated with the degree of information about PPM therapy (p = 0.001), dependency on the device (p = 0.001), social difficulties due to the device (p = 0.001), and whether participants believed that the device prevents disease deterioration (p = 0.004). Specifically, patients who were well informed about PPM therapy (median: 72), those who did not feel dependency on the device (median: 76), those who did not face social difficulties due to the device (median: 72), and those who did not believe that the device prevents disease deterioration (median: 72) had a better emotional well-being. Also, social functioning **(Table 4)** was statistically significantly associated with the degree of information about PPM therapy (p = 0.002), whether patients attended their scheduled follow-up (p = 0.003), the patient’s dependency on the device (p = 0.002), social difficulties due to the device (p = 0.001), and whether participants believed that the device prevents disease deterioration (p = 0.001). Additionally, older patients had worse social functioning (rho = −0.235). Patients well informed about PPM therapy (median: 75), those who regularly attended the scheduled follow-up (median: 75), those who did not feel dependency on the device (median: 75), those who did not face social difficulties due to the device (median: 75), and those who did not believe that the device prevents disease deterioration (median: 75) had better social functioning.

Pain **(Table 5)** was found to be statistically significantly associated with the degree of information about the patient’s family (p = 0.001), social difficulties due to the device (p = 0.001), and whether the patient believed that the device prevents disease deterioration (p = 0.005). More specifically, patients whose family was well informed about PPM therapy (median: 90), those who did not face social difficulties due to the device (median: 77.5), and those who did not believe that the device prevents disease deterioration (median: 77.5) had a better QoL. Additionally, older patients had worse social functioning (rho = −0.215). General health **(Table 5)** was statistically significantly associated with the degree of information about PPM therapy (p = 0.001), family support (p = 0.036), whether patients attended their scheduled follow-up (p = 0.016), social difficulties due to the device (p = 0.004), and whether participants believed that the device prevents disease deterioration (p = 0.001). Those patients who reported they were well informed about PPM therapy (median: 55), those with very supportive family members (median: 52), those who regularly attended the follow-up (median: 55), those who did not face social difficulties due to the device (median: 55), and those who did not believe that the device prevents disease deterioration (median: 60) had better general health.

### Effect of characteristics on patients’ quality of life

Multiple linear regression was then performed with the patient QoL subscales as dependent variables in order to estimate the effect of patients’ characteristics and their anxiety/depression (independent factors).

Regarding patients’ characteristics **(Tables 6–8)**, a one-year increase in age indicates a 0.9-point decrease in physical and emotional roles (95% CI: −1.6 to 0.3; p = 0.005 and 95% CI: −1.5 to 0.2; p = 0.009, respectively), a 0.7-point decrease in social functioning (95% CI: −1.1 to 0.3; p = 0.002), and a 0.6-point decrease in pain (95% CI: −1.1 to 0.1; p = 0.013), leading to a worse QoL. Patients sufficiently informed about PPM therapy had a 19.1-point worse physical role (95% CI: −34.4 to 3.9; p = 0.14), a 9.1-point worse emotional well-being (95% CI: −14.9 to 3.3; p = 0.002), and a 13.1-point worse pain score (95% CI: −23.8 to 2.5; p = 0.016) than those who were well informed. Likewise, patients who were a little or not at all informed had a 10.7-point worse emotional well-being than those who were well informed (95% CI: −19.1 to 2.3; p = 0.013). Those who did not attend their scheduled follow-up had 21.7- and 22.2-point worse physical and emotional roles, respectively, than those who regularly attended their follow-up (95% CI: −42.4 to 1.1; p = 0.039 and 95% CI: −44.0 to 0.4; p = 0.046, respectively). Those who did not express the belief that the device prevents disease deterioration had a 31.8-point better emotional role and 18.9-point improved social functioning, respectively, than those who did (95% CI: 10.7–52.9; p = 0.003 and 95% CI: 5.6–32.2; p = 0.006, respectively). Moreover, patients who did not feel dependent on the device had 16.1-, 15.7-, and 13.7-point better energy, emotional well-being, and social functioning, respectively (95% CI: 7.9–24.3, p = 0.001; 95% CI: 8.6–22.7, p = 0.001; and 95% CI: 2.6–24.8, p = 0.001, respectively).

## Discussion

According to the reported results, PPM patients had the lowest SF-36 scores in physical role (35.4 ± 41.1 points) and physical functioning (22.5 ± 10.7 points). Similarly, a recent study of 88 patients (aged 64.3 ± 13 years) living with their devices for at least one month showed the lowest rates in the physical health and physical functioning domains.^13^ Relevant studies have also revealed a low QoL in physical functioning.^14,15^ Differences in QoL were observed over time, with all SF-36 scores gradually declining postimplantation, but they remained improved relative to the pre-implantation ones throughout the 7.5-year observation period.^16^ A prior study by Fleischmann et al.^17^ [Mode Selection Trial (MOST) study], which examined 2,010 patients during a four-year follow-up period, showed that scores of role functioning and mental health remained above the pre-implantation ones, whereas scores of physical domains were comparable to the pre-implantation values.

In terms of descriptive results, 18% of participants reported to be only a little or not informed about PPM therapy and 15.3% believed that the device would prevent disease progression. Similarly, in a relevant study, the majority of patients with implanted electronic devices (mean age: 64 years, 33% women, 39% New York Heart Association class II) believed that their devices would forestall further disease deterioration.^11^ Overestimating the potential benefits of cardiac devices on disease progression is an obstruction in care and in treatment adherence. More strikingly, technological advances may reinforce the belief that new and complex innovations may soon be available to forestall death.^11^

Relatively, the majority (88.6%) among 70 PPM patients (61.71 ± 12.42 years, 60% men, duration of implantation: 2.9 ± 5.21 years) acknowledged the device as a cure for their heart disease, while 25.7% believed that the device would be removed if they remained symptom-free.^18^ Likewise, among 250 PPM patients, 94.8% erroneously believed that device implantation was enough to treat arrhythmia, and 17.6% continued smoking, 85.3% consumed alcohol occasionally, and 44.4% did not perform any exercise.^2^ Misunderstandings arising from outdated information and popular notions contribute to unrealistic expectations, which in turn indirectly influence the QoL.^2,3,19^ Therefore, it remains imperative to shed more light on patients’ misconceptions about the role of cardiac devices in disease management. Based on the findings presented, it is suggested that understanding these perceptions/misconceptions is fundamental when developing interventions that place the right emphasis on device utility and enhance care dialogue. After an educational intervention, PPM patients acknowledged their illness as a chronic condition that is responsive to treatment and influenced by personal behavior.^19^

Furthermore, well-informed patients had a better QoL in physical and emotional roles, social functioning, and general health. Elaborate information is recommended as an integral part of treatment.^20^ Interestingly, well-informed patients are collaborative with clinicians, are more involved in their care, and avoid problematic and unreliable treatment practices, which in turn positively affect their QoL by improving clinical outcomes.^20^ There is a positive correlation between the knowledge of participants and their QoL.^19^

Better QoL was observed among patients who reported having a supportive family (in both an emotional capacity and regarding general health) and those having a well-informed family (in pain). Possibly, family support provides a sense of security to the individuals, which enhances their confidence to overcome difficulties, thus increasing their QoL. Although many different definitions for support are provided in the literature, they all share common characteristics and imply any type of positive interaction or helpful behavior provided to a person in need.^21–23^ Subjects with spouses as their main caregivers have better improvement in QoL after PM implantation.^23^ Support provides a relaxing environment that enables recipients to accept the device,^22^ although it is negatively associated with sleep quality.^21^ Also, an increase in social support either by significant ones, family, or friends leads to a decrease in state and trait anxiety.^24^

An encouraging finding of this study is that patients who attended the scheduled follow-up as recommended by health professionals had a better QoL in physical and emotional roles, social functioning, and general health. A possible explanation for this finding is that follow-up visits offer individualized care, communication, and counseling, which in turn enable patients to achieve the best possible QoL within the limitations of the disease process. During follow-up, PPM recipients have the opportunity to communicate and express their perceptions about several aspects such as benefits and burdens of device therapy, changes in health status, context of illness, and potential clinical outcomes. At the same time, health professionals have the advantageous ability to identify their values and goals regarding health care and incorporate them into participatory planning and decision-making.^25^ A follow-up visit includes evaluations of the device function, optimization of the system function, exploration of PM complications, provision of support or guidance, and scheduling of the next visit.^26^ Moreover, PM follow-up visits provide important data about patients’ clinical status, such as heart rate histograms, heart rate variability, arrhythmia episodes, and patient activity.^9^

Furthermore, participants reporting no social difficulties due to the device had a better QoL in terms of emotional well-being, social functioning, pain, and general health, while those reporting no dependency on the device had a better QoL in the areas of energy/fatigue, emotional well-being, and social functioning. Possibly, these recipients had already accepted the device and rejected dependency, which is associated with negative feelings and vulnerability. A relevant study conducted by De Bardi et al.^22^ of 62 patients (median age: 76.5 years) reported increases in support, acceptance of the cardiac device, and QoL after 30 days.

It should be stressed that 29.3% of participants declared a high dependency on their devices. This finding emphasizes the need to evaluate patients’ perspectives that exert a significant influence on cardiac disease, such as changing health behaviors, following recommended treatments, and rehabilitation.^24^ More dependency-related issues are anticipated to emerge at the forefront of clinical practice along with PM technology progression. Technological advances in electronic device implantation are inevitably associated with several improvements in health and longevity but simultaneously induce a degree of care of advanced complexity. Although, nowadays, health professionals are overqualified, dependency still remains an issue poorly understood from both research and clinical perspectives as it is not typically verbalized by them either as part of care or during collaboration with colleagues.^27^

Last, but not least, older patients had worse physical and emotional roles, social functioning, and pain. On the contrary, a prior study by Malm et al.^28^ showed a better QoL in individuals aged between 65 and 84 years, those who were cohabiting, those who had their own dwelling, and those who had a PM for three years or less. The elderly may better adapt to their disease as they have already worked, raised their families, or have lower expectations regarding their remaining lifespan.^29^ On the other hand, young individuals (aged 18–29 years) may experience insecurity about their physical appearance, uncertainty about the future, and limited support.^30^ Therefore, the challenges of living with a PPM is an issue of paramount importance among all age groups, especially with regard to QoL.

### Limitations of this study

The method used in the present study was convenience sampling, and, therefore, the study population is not representative of all patients in Greece nor elsewhere in the world. Moreover, it was a cross-sectional study, thus not allowing the emergence of a causal relation between QoL and patients’ self-reported characteristics. Moreover, data for this study were collected during a single interview two years after device implantation. The lack of a preoperative evaluation may be questioned; however, available data have systematically shown that preoperative QoL scores are lower relative to the postoperative ones.

In terms of our small sample size, it could be possible that a large randomized trial might have possibly detected more significant statistical differences. Further studies are needed to confirm these findings as more evidence is required.

The strength of this study is the use of the SF-36 instrument, which is easy to interpret and well researched as it has been widely applied in a large number of patients. This instrument may permit comparisons between populations all over the world. Additionally, this widely accepted instrument may reveal in which areas interventions should be focused for managing device-implanted patients.

## Conclusions

Our results reveal that QoL was associated with the degree of information of patients and their family about PPM, attendance at follow-up, the belief that the device prevents disease deterioration, feelings of dependency on the device, and social difficulties attributed to the device.

In terms of pacing therapy, embracing device technology is not solely enough to improve QoL. Therefore, it is important to consider the impact of patients’ perceptions in addition to cardiac pacing and identify particular domains in which interventions may be developed and applied.

## Figures and Tables

**Table 1:** Sample Description (N = 150)

Informed about PPM therapy	
Well, n (%)	53 (35.3%)
Sufficiently, n (%)	70 (46.7%)
A little, n (%)	22 (14.7%)
Not at all, n (%)	5 (3.3%)
Attended scheduled follow-up	
Regularly, n (%)	43 (28.7%)
Sufficiently, n (%)	78 (52.0%)
A little, n (%)	27 (18.0%)
Not at all, n (%)	2 (1.3%)
Dependency on the device	
High, n (%)	44 (29.3%)
Moderate, n (%)	65 (43.3%)
A little, n (%)	38 (25.3%)
Not at all, n (%)	3 (2.0%)
Does the device prevent disease deterioration?	
Yes, n (%)	23 (15.3%)
No, n (%)	69 (46.0%)
Possibly, n (%)	58 (38.7%)
Family informed about PPM therapy	
Well, n (%)	61 (40.7%)
Sufficiently, n (%)	78 (52.0%)
A little, n (%)	10 (6.7%)
Not at all, n (%)	1 (0.7%)
Is your family supportive?	
Very, n (%)	72 (48.0%)
Sufficiently, n (%)	67 (44.7%)
A little, n (%)	8 (5.3%)
Not at all, n (%)	3 (2.0%)
Relationship with nursing staff	
Very good, n (%)	114 (76.0%)
Good, n (%)	36 (24.0%)
Relationship with medical staff	
Very good, n (%)	109 (72.7%)
Good, n (%)	41 (27.3%)
Social difficulties experienced due to the device	
Very, n (%)	4 (2.7%)
Enough, n (%)	11 (7.3%)
A little, n (%)	44 (29.3%)
Not at all, n (%)	91 (60.7%)

PPM: permanent cardiac pacemaker.

**Table 2:** Levels of Patients’ QoL (N = 150)

QoL Dimension*	Mean (SD)
Physical functioning	22.5 (10.7) points
Physical role	35.4 (41.1) points
Emotional role	38.9 (45.2) points
Energy/fatigue	57.1 (20.1) points
Emotional well-being	66.5 (18.8) points
Social functioning	61.7 (27.0) points
Pain	63.8 (28.5) points
General health	48.0 (20.1) points
Physical health	42.9 (19.5) points
Mental health	54.1 (26.6) points

SD: standard deviation: QoL: quality of life.

*All dimensions scored using a range of zero to 100 points.

**Table 3:** Association Between Patients’ Characteristics and QoL in Physical Functioning, Physical Role, and Emotional Role Dimensions

	Physical Functioning		Physical Role		Emotional Role	
	Median (IQR)	p-value	Median (IQR)	p-value	Median (IQR)	p-value
Informed about PPM therapy		0.586		0.005*		0.004*
Well	22 (14–28)		50 (0–100)		67 (0–100)	
Sufficiently	21 (14-26)		0 (0–50)		0 (0–100)	
A little/not at all	16 (5–28)		0 (0–25)		0 (0–67)	
Family informed about therapy		0.321		0.076		0.070
Well	22 (14–28)		25 (0–100)		33 (0–100)	
Sufficiently	20.5 (10–26)		0 (0–75)		0 (0–100)	
A little/not at all	15 (14–26)		0 (0–0)		0 (0–0)	
Is your family supportive?		0.636		0.053		0.023*
Very	21 (11–26)		12.5 (0–75)		33 (0–100)	
Sufficiently	21 (14–27)		0 (0–100)		0 (0–100)	
A little/not at all	25 (14–30)		0 (0–0)		0 (0–0)	
Attended scheduled follow-up		0.570		0.015*		0.014*
Regularly	25 (14–28)		50 (0–100)		67 (0–100)	
Sufficiently	21 (10–26)		0 (0–75)		0 (0–100)	
A little/not at all	21 (11–27)		0 (0–50)		0 (0–33)	
Relationship with nursing staff		0.087		0.343		0.090
Very good	20.5 (8–27)		0 (0–100)		33 (0–100)	
Good	22 (16–26.5)		0 (0–75)		0 (0–83)	
Relationship with medical staff		0.080		0.543		0.449
Very good	21 (8–27)		0 (0–100)		0 (0–100)	
Good	22 (16–27)		0 (0–75)		0 (0–100)	
Dependency on the device		0.720		0.070		0.056
Very	21 (16–26)		0 (0–62.5)		0 (0–100)	
Enough	22 (11–26)		0 (0–50)		0 (0–100)	
A little/not at all	21 (8–35)		50 (0–100)		33 (0–100)	
Social difficulties due to the device		0.104		0.521		0.397
Very/enough	24 (10–26)		50 (0–50)		0 (0–67)	
A little	16 (5–26.5)		0 (0–87.5)		0 (0–100)	
Not at all	22 (15–28)		0 (0–100)		0 (0–100)	
Does the device prevent disease deterioration?		0.845		0.005*		0.001*
Yes	22 (15–27)		0 (0–0)		0 (0–0)	
No	21 (14–26)		50 (0–100)		33 (0–100)	
Possibly	20.5 (10–30)		0 (0–50)		0 (0–100)	
	**Spearman’s rho**		**Spearman’s rho**		**Spearman’s rho**	
Age (years)	−0.163	0.067	−0.230	0.015*	−0.220	0.017*

IQR: interquartile range; PPM: permanent cardiac pacemaker.

*Statistically significant.

**Table 4:** Association Between Patients’ Characteristics and QoL in Energy/Fatigue, Emotional Well-being, and Social Functioning Dimensions

	Energy/Fatigue		Emotional Well-being		Social Functioning	
	Median (IQR)	p-value	Median (IQR)	p-value	Median (IQR)	p-value
Informed about PPM therapy		0.070		0.001*		0.002*
Well	60 (35–70)		72 (64–84)		75 (50–100)	
Sufficiently	60 (45–70)		64 (48–76)		62.5 (50–100)	
A little/not at all	35 (20–70)		44 (40–56)		37.5 (12.5–50)	
Family informed about PPM therapy		0.464		0.355		0.011*
Well	60 (50–70)		72 (48–84)		75 (37.5–100)	
Sufficiently	60 (35–70)		64 (44–80)		50 (37.5–87.5)	
A little/not at all	45 (30–65)		64 (52–72)		25 (12.5–50)	
Is your family supportive?		0.196		0.210		0.061
Very	60 (35–70)		72 (44–84)		62.5 (25–100)	
Sufficiently	60 (40–70)		64 (48–76)		62.5 (50–87.5)	
A little/Not at all	30 (20–65)		64 (40–68)		50 (25–50)	
Attended scheduled follow-up		0.479		0.071		0.003*
Regularly	60 (30–70)		68 (52–84)		75 (25–100)	
Sufficiently	60 (50–70)		68 (48–80)		62.5 (50–100)	
A little/not at all	55 (30–70)		56 (40–68)		50 (25–50)	
Relationship with nursing staff		0.377		0.159		0.754
Very good	60 (35–70)		68 (48–84)		56.25 (37.5–100)	
Good	57.5 (37.5–80)		64 (44–76)		50 (37.5–81.25)	
Relationship with medical staff		0.257		0.656		0.963
Very good	60 (35–70)		68 (48–80)		50 (37.5–100)	
Good	60 (40–80)		64 (48–80)		50 (37.5–87.5)	
Dependency on the device		0.006*		0.001*		0.002*
High	60 (20–70)		66 (42–84)		75 (37.5–93.75)	
Moderate	55 (35–60)		60 (40–68)		50 (25–75)	
A little/not at all	65 (55–75)		76 (64–84)		75 (50–100)	
Social difficulties due to the device		0.062		0.001*		0.001*
Very/enough	45 (20–80)		64 (40–80)		50 (12.5–87.5)	
A little	45 (30–65)		56 (40–68)		50 (25–68.75)	
Not at all	60 (50–70)		72 (52–84)		75 (50–100)	
Does the device prevent disease deterioration?		0.010*		0.004*		0.001*
Yes	55 (20–65)		64 (40–72)		25 (12.5–75)	
No	65 (50–75)		72 (56–84)		75 (62.5–100)	
Possibly	55 (30–65)		64 (44–72)		50 (25–50)	
	**Spearman’s rho**		**Spearman’s rho**		**Spearman’s rho**	
Age (years)	−0.125	0.127	−0.110	0.182	−0.235	0.015*

IQR: interquartile range; PPM: permanent cardiac pacemaker.

*Statistically significant.

**Table 5:** Association Between Patients’ Characteristics and QoL in Pain and General Health Dimensions

	Pain		General Health	
	Median (IQR)	p-value	Median (IQR)	p-value
Informed about PPM therapy		0.060		0.001*
Well	67.5 (35–100)		55 (40–70)	
Sufficiently	67.5 (45–100)		50 (35–65)	
A little/not at all	32.5 (10–77.5)		30 (25–52)	
Family informed about PPM therapy		0.001*		0.023*
Well	90 (47.5–100)		55 (40–70)	
Sufficiently	56.25 (22.5–77.5)		50 (30–65)	
A little/not at all	45 (45–67.5)		30 (27–40)	
Is your family supportive?		0.238		0.036*
Very	72.5 (36.25–100)		52 (40–63.5)	
Sufficiently	57.5 (22.5–77.5)		50 (30–70)	
A little/not at all	67.5 (45–77.5)		30 (20–32)	
Attended scheduled follow-up		0.099		0.016*
Regularly	77.5 (37.5–100)		55 (40–62)	
Sufficiently	67.5 (32.5–100)		50 (35–70)	
A little/not at all	57.5 (32.5–77.5)		32 (25–55)	
Relationship with nursing staff		0.596		0.392
Very good	67.5 (32.5–100)		50 (30–62)	
Good	62.5 (45–100)		53.5 (32–67.5)	
Relationship with medical staff		0.500		0.338
Very good	67.5 (32.5–100)		50 (30–62)	
Good	67.5 (45–100)		52 (32–65)	
Dependency on the device		0.052		0.073
High	57.5 (32.5–95)		47.5 (27.5–67.5)	
Moderate	55 (22.5–77.5)		45 (30–55)	
A little/not at all	100 (57.5–100)		55 (45–70)	
Social difficulties due to the device		0.001*		0.004*
Very/enough	45 (22.5–100)		40 (32–65)	
A little	45 (22.5–67.5)		40 (22.5–55)	
Not at all	77.5 (45–100)		55 (35–70)	
Does the device prevent disease deterioration?		0.005*		0.001*
Yes	45 (22.5–100)		40 (32–60)	
No	77.5 (55–100)		60 (42–75)	
Possibly	51.25 (32.5–77.5)		40 (25–55)	
	**Spearman’s rho**		**Spearman’s rho**	
Age (years)	−0.215	0.018*	−0.091	0.270

IQR: interquartile range; PPM: permanent cardiac pacemaker.

*Statistically significant.

**Table 6:** Impact of Patients’ Characteristics on QoL in Physical Functioning, Physical Role, and Emotional Role Dimensions

	Physical Functioning		Physical Role		Emotional Role	
	β Coefficient (95% CI)	p-value	β Coefficient (95% CI)	p-value	β Coefficient (95% CI)	p-value
Age (years)	-		−0.9 (−1.6 to 0.3)	0.005*	−0.9 (−1.5 to −0.2)	0.009*
Informed about PPM therapy						
Well	-		Reference		Reference	
Sufficiently	-		−19.1 (−34.4 to −3.9)	0.014*	−14.2 (−30.4 to 2.0)	0.085
A little/not at all	-		−6.7 (−28.0 to 14.6)	0.537	−0.3 (−23.2 to 22.6)	0.980
Family support						
Very	-		-		Reference	
Sufficiently	-		-		10.1 (−5.3 to 25.4)	0.196
A little/not at all	-		-		−8.5 (−38.8 to 21.7)	0.579
Attended scheduled follow-up						
Regularly	-		Reference		Reference	
Sufficiently	-		−21.3 (−36.7 to −5.9)	0.007*	−13.6 (−29.7 to 2.4)	0.096
A little/not at all	-		−21.7 (−42.4 to −1.1)	0.039*	−22.2 (−44.0 to −0.4)	0.046*
Does the device prevent disease deterioration?						
Yes	-		Reference		Reference	
No	-		12.2 (−7.9 to 32.4)	0.233	31.8 (10.7 to 52.9)	0.003*
Possibly	-		−0.5 (−20.7 to 19.7)	0.958	28.8 (7.7 to 49.9)	0.008*

CI: confidence interval; PPM: permanent cardiac pacemaker.

*Statistically significant.

**Table 7:** Impact of Patients’ Characteristics on QoL in Energy/Fatigue, Emotional Well-being, and Social Functioning Dimensions

	Energy/Fatigue		Emotional Well-being		Social Functioning	
	β Coefficient (95% CI)	p-value	β Coefficient (95% CI)	p-value	β Coefficient (95% CI)	p-value
Age (years)	-		-		−0.7 (−1.1 to −0.3)	0.002*
Informed about PPM therapy						
Well	-		Reference		Reference	
Sufficiently	-		−9.1 (−14.9 to −3.3)	0.002*	5.6 (−3.8 to 15.0)	0.240
A little/not at all	-		−10.7 (−19.1 to −2.3)	0.013*	6.9 (−6.5 to 20.3)	0.308
Family informed about PPM therapy						
Well	-		-		Reference	
Sufficiently	-		-		2.9 (−6.4 to 12.1)	0.545
A little/not at all	-		-		−11.9 (−29.5 to 5.8)	0.185
Attended scheduled follow-up						
Regularly	-		-		Reference	
Sufficiently	-		-		−3.3 (−13.4 to 6.9)	0.528
A little/not at all	-		-		−8.5 (−21.5 to 4.5)	0.200
Dependency on the device						
High	Reference		Reference		Reference	
Moderate	6.9 (−0.5 to 14.4)	0.068	2.1 (−4.4 to 8.6)	0.527	−0.4 (−10.7 to 9.9)	0.944
A little/not at all	16.1 (7.9 to 24.3)	0.001*	15.7 (8.6 to 22.7)	0.001*	13.7 (2.6 to 24.8)	0.016*
Social difficulties due to the device						
Very/enough	-		Reference		Reference	
A little	-		−7.5 (−17.7 to 2.7)	0.147	−7.8 (−24.0 to 8.4)	0.340
Not at all	-		−1.2 (−10.6 to 8.1)	0.791	2.3 (−13.3 to 17.9)	0.771
Does the device prevent disease deterioration?						
Yes	Reference		Reference		Reference	
No	4.1 (−5.2 to 13.3)	0.390	−0.4 (−8.8 to 8.0)	0.928	18.9 (5.6 to 32.2)	0.006*
Possibly	1.7 (−7.6 to 10.9)	0.719	1.8 (−6.8 to 10.3)	0.686	5.6 (−8.1 to 19.3)	0.421

CI: confidence interval; PPM: permanent cardiac pacemaker.

*Statistically significant.

**Table 8:** Impact of Patients’ Characteristics on QoL in Pain and General Health Dimensions

	Pain		General Health	
	β Coefficient (95% CI)	p-value	β Coefficient (95% CI)	p-value
Age (years)	−0.6 (−1.1 to −0.1)	0.013*	-	
Informed about PPM therapy				
Well	-		Reference	
Sufficiently	-		0.4 (−7.4 to 8.3)	0.914
A little/not at all	-		−2.6 (−13.9 to 8.6)	0.643
Family informed about PPM therapy				
Well	Reference		Reference	
Sufficiently	−13.1 (−23.8 to −2.5)	0.016*	−2.6 (−13.2 to 8.0)	0.625
A little/not at all	−11.3 (−32.6 to 10.1)	0.299	0.7 (−16.9 to 18.3)	0.938
Is your family supportive?				
Very	-		Reference	
Sufficiently	-		2.1 (−8.4 to 12.7)	0.689
A little/not at all	-		−14.4 (−31.6 to 2.8)	0.100
Attended scheduled follow-up				
Regularly	-		Reference	
Sufficiently	-		−3.7 (−11.7 to 4.3)	0.367
A little/not at all	-		−4.4 (−15.3 to 6.6)	0.433
Social difficulties due to the device				
Very/enough	Reference		Reference	
A little	−1.2 (−20.3 to 17.8)	0.899	−4.0 (−17.3 to 9.3)	0.549
Not at all	13.3 (−5.1 to 31.7)	0.155	2.0 (−10.3 to 14.4)	0.744
Does the device prevent disease deterioration?				
Yes	Reference		Reference	
No	6.2 (−9.4 to 21.8)	0.432	5.4 (−5.3 to 16.2)	0.319
Possibly	0.2 (−15.8 to 16.2)	0.978	−6.2 (−17.2 to 4.7)	0.263

CI: confidence interval; PPM: permanent cardiac pacemaker.

*Statistically significant.
